# Can we assess the advancements of gallbladder cancer using red blood cell distribution width?

**DOI:** 10.1097/MD.0000000000018364

**Published:** 2019-12-20

**Authors:** Youjun Xie, Lingling Zhang, Lingling Zhan

**Affiliations:** a Department of Clinical Laboratory, First Affiliated Hospital of Guangxi Medical University; b Department of Clinical Laboratory, Children's Hospital, Maternal and Child Health Hospital of Guangxi Zhuang Autonomous Region, Nanning, China.

**Keywords:** gallbladder cancer, inflammation, metastasis, progression, red cell distribution width

## Abstract

Gallbladder cancer (GBC) is a rare biliary malignancy. The relationship between red blood cell distribution width (RDW) and cancer prognosis has been confirmed by many studies, however, the relationship between RDW and gallbladder cancer is rarely reported. Therefore, we aimed to assess the correlation between RDW and the advancements of GBC in this study.

A retrospective study was performed on 108 GBC patients and 119 age and gender-matched individuals who were admitted to the First Affiliated Hospital of Guangxi Medical University from January 2012 to December 2018.

The GBC patients had significantly higher RDW(%) levels compared to the healthy controls group (15.7 ± 2.4 vs 13.5 ± 0.6; *P* = .000). In addition, GBC patients with stage III+IV had higher levels of RDW(%) than stage I+II (16.1 ± 2.5 vs 14.9 ± 2.0, *P* = .011). Correlation analysis showed that RDW had positive correlations with TNM stage (correlation coefficient = 0.302, *P* = .002). The cut-off value of RDW was observed to be 14.5% in patients with GBC (area under the curve = 0.757, 95% confidence interval = 0.677–0.838, *P* = .000). Univariate logistic regression and multivariate logistic regression analysis showed that RDW was an independent risk factor for GBC lymph node metastasis.

Our results suggest that elevated levels of RDW are independently associated with GBC patients and may serve as potential markers for the advancements of GBC.

## 1 Introduction

Gallbladder carcinoma (GBC), a malignant tumor originating from the epithelium of the gallbladder, is the most common tumor in gallbladder malignancies.^[1,2]^ The overall incidence of GBC worldwide is approximately 2/100,000. However, the incidence varies by geographic region. In Northern Europe, the incidence of GBC is less than 1/100,000, among Native Americans, its incidence is as high as 23/100,000.^[3,4]^ As the shortest survival malignant tumor in biliary tract tumors, GBC is highly invasive and has a poor prognosis.^[5–9]^ There is a lack of specific performance in early GBC. Most patients have been accompanied by local infiltration or distant metastasis at the time of diagnosis, thus miss the best time for treatment.^[2,10,11]^ Thus, it is critical for early diagnosis of GBC.

Red blood cell distribution width (RDW) is one of the routine indicators of whole blood cell count examination, reflecting the heterogeneity of red blood cell volume.^[12]^ Similar to the neutrophil to lymphocyte ratio (NLR) and platelet to lymphocyte ratio (PLR), RDW is also considered to be one of the markers of inflammation. Studies have found that preoperative NLR, PLR and serum carbohydrate antigen 19–9 (CA19–9) levels are associated with poor prognosis of GBC.^[13,14]^ However, the relationship between RDW and GBC is rarely reported. Hence, we intend to explore the significance of RDW in the advancements of GBC in this study.

## 2 Methods

### 2.1 Patients

Approval for the study was granted by the Ethical Committee of the First Affiliated Hospital of Guangxi Medical University. Patients with hepatitis, malignant tumors, blood pressure, cardiovascular disease, diabetes, kidney disease, acute inflammation, blood diseases, recent blood transfusions, and venous thrombosis for more than six months were excluded from the study. In this retrospective study, 108 patients with GBC admitted to the First Affiliated Hospital of Guangxi Medical University (Nanning, China) from January 2012 to December 2018 were included.119 age- and gender-matched individuals with a regular physical examination at our hospital was used as a control. All patients with GBC underwent histological analysis and were classified according to pathology and/or cytology: 20(18.5%) were TNM I, 18(16.7%) were TNM II, 17(15.7%) were TNM III and 53(49.1%) were TNM IV. In addition, according to the metastatic site, 39(36.1%) had untransferred GBC, 17(15.7%) had liver metastases, 44(40.7%) had liver metastases with distant metastases, and 8(7.4%) had extensive abdominal metastasis.

### 2.2 Methods

The 2 ml of venous blood was taken from all subjects in the morning, and GBC patients were collected before any treatment. All blood specimens were placed in EDTA-K2 tubes. White blood cell counts (WBC), hemoglobin (Hb), neutrophil counts (NEU), lymphocyte counts (LYM), mean red blood cell volume (MCV), RDW and platelet counts (PLT) were determined using a Beckman Coulter LH780 hematology analyzer (Beckman Coulter, Brea, CA) within 30 minutes after blood collection.

### 2.3 Statistical analysis

SPSS software (version 22.0; Chicago, IL) was used for statistical analysis. A *P* value of less than .05 was considered to be statistically different. Measurements that conformed to the normal distribution were compared using two independent sample t tests, expressed as mean ± standard deviation; the Mann Whitney *U* test was used to compare non-normally distributed data, expressed as median (interquartile range). One-way ANOVA was used to compare multiple groups. Based on the type of data distribution, correlation analysis was performed using Pearson approach or Spearman approach. Receiver operating characteristics curve (ROC) was used to measure the performance of RDW in estimating GBC.

## 3 Results

Clinical data and laboratory measurements of all participants are presented in Table 1. There were significant differences in WBC, Hb, NEU, LYM, MCV, RDW, PLT, MPV, and PDW between patients with GBC and healthy individuals (*P* < .05). Of note, the RDW (%) level of GBC patients was higher than that of healthy controls (15.7 ± 2.4 vs 13.5 ± 0.6; *P* = .000).

**Table 1:**
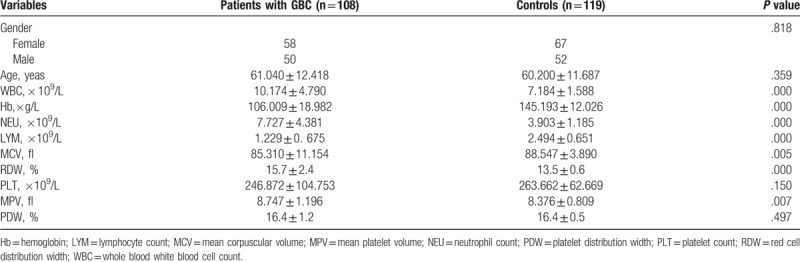
Comparison of demographic and laboratory parameters of patients with GBC and healthy controls.

**Figure 1:**
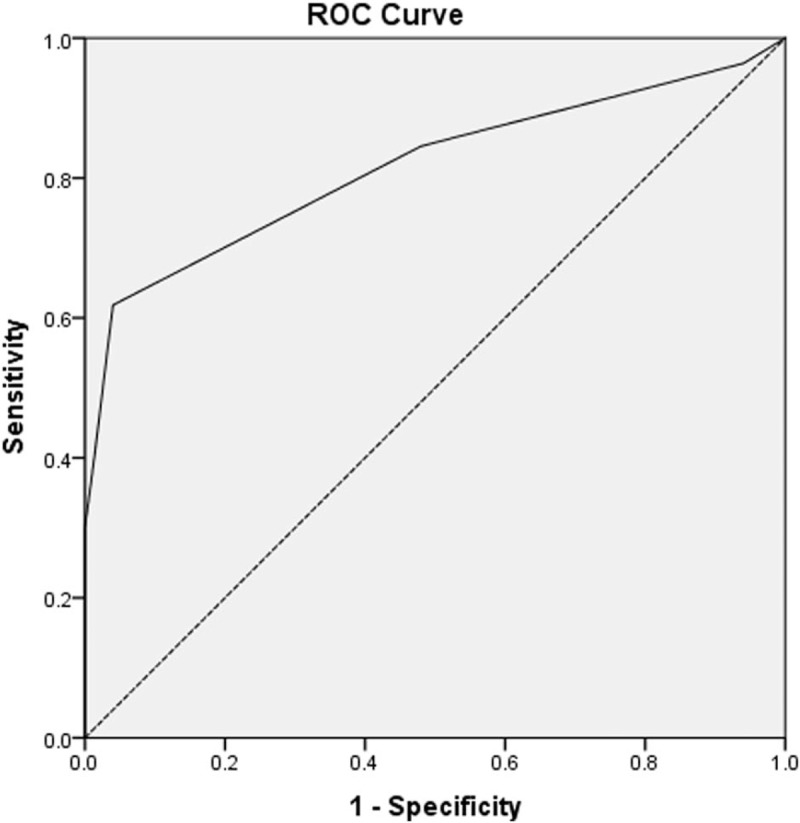
Diagnostic performance of the RDW in detecting GBC. GBC = gallbladder carcinoma; RDW = red blood cell distribution width.

Receiver-operating characteristic analysis was used to evaluate the AUC of RDW in GBC. ROC analysis indicated that RDW had a sensitivity of 57.7% and a specificity of 89.0% for predicting GBC progression (AUC = 0.757, 95% confidence interval: 0.677–0.838, *P* = .000). An optimal cut-off value of 14.5% was observed for RDW in patients with GBC (Fig. 1). To further explore the effect of RDW levels on clinical stage and laboratory parameters of GBC patients, we grouped and analyzed RDW = 14.5% as the critical value. Of the 108 GBC patients, 40 (37%) had RDW levels below 14.5%, while 68 (63%) had RDW levels above 14.5%. Statistical analysis showed that there were significant differences between the 2 groups of GBC patients in terms of Hb, MPV, and TNM stage (all *P* < .05) (Table 2).

**Table 2:**
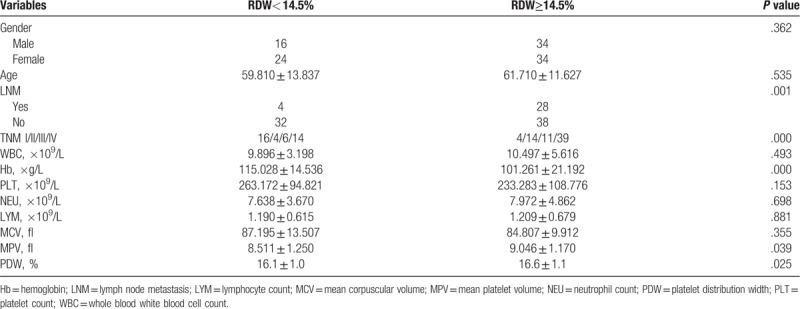
Clinical pathological factors of patients with RDW < 14.5% compared with RDW ≥ 14.5%.

**Figure 2:**
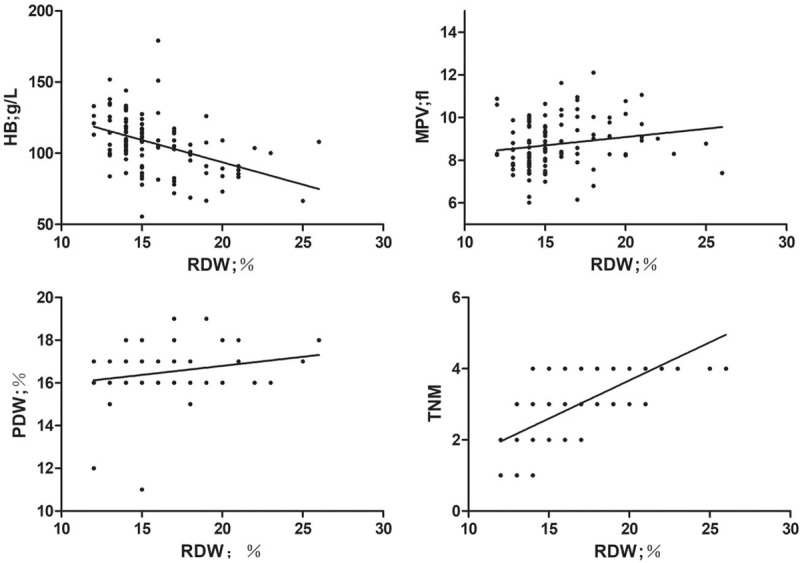
Correlation analyses between RDW and Hb, MPV, PDW and TNM stages. Hb = hemoglobin; MPV = mean platelet volume; PDW = platelet distribution width; RDW = red cell distribution width; TNM = tumor node metastasis.

In the correlation analysis, we assessed the association of RDW with other laboratory parameters and disease stages in GBC patients. Statistical analysis showed that RDW was negatively correlated with Hb level (correlation coefficient = −0.532, *P* = .000). However, RDW was positively correlated with PDW (correlation coefficient = 0.244, *P* = .000) and MPV (correlation coefficient = 0.291, *P* = .000). Interestingly, RDW was found to be positively associated with tumor TNM stage (correlation coefficient = 0.302, *P* = .002) (Fig. 2).

**Figure 3:**
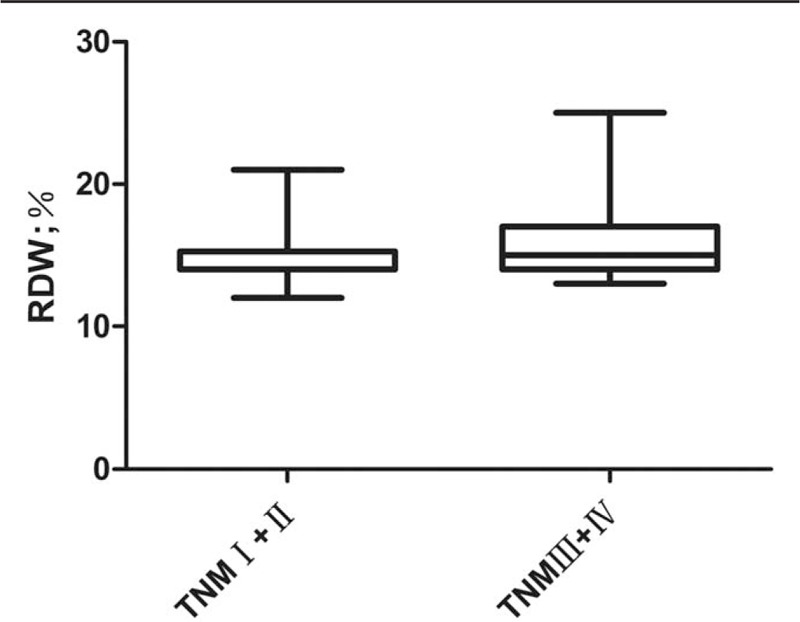
Comparison of RDW in TNM staging subgroup. RDW = red blood cell distribution width; TNM = tumor node metastasis.

**Figure 4:**
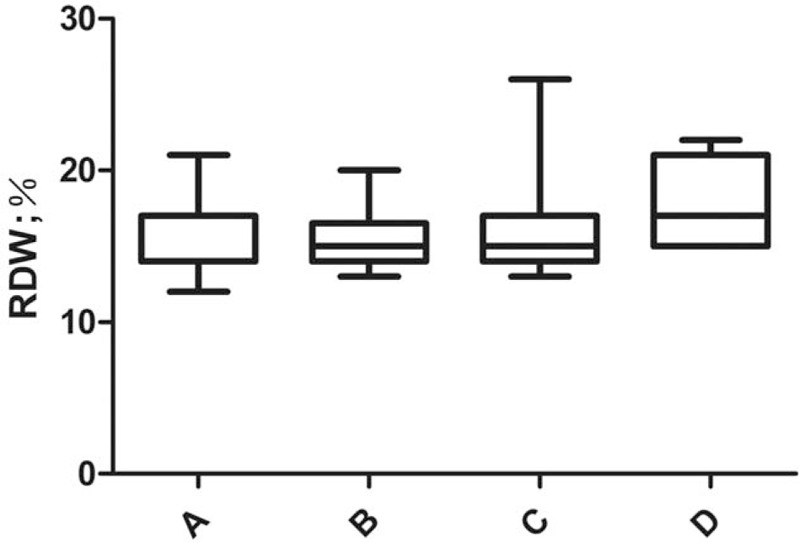
Comparison of RDW at different sites of metastasis. A = no metastasis; B = liver metastasis; C = liver metastasis with extrahepatic metastasis; D = extensive peritoneal metastasis; RDW = red blood cell distribution width.

We compared RDW (%) with different TNM staging and found that RDW levels were significantly higher in patients with stage III+IV (n = 38) than in stage I+II (n = 70) (16.1 ± 2.5 vs 14.9 ± 2.0, *P* = .011; Fig. 3). One-way ANOVA showed that the RDW (%) values of the non-metastatic group, liver metastasis group, liver metastasis combined with extrahepatic metastasis group and extensive abdominal metastasis group were 15.1 ± 2.1, 15.6 ± 2.1, 16.1 ± 2.9 and 18.0 ± 3.2, respectively, with statistically significant differences (*P* = .041). Patients with abdominal metastases had the highest RDW (Fig. 4).

Univariate logistic regression and multivariate logistic regression analysis showed that RDW was an independent risk factor for GBC lymph node metastasis (Table 3).

**Table 3:**
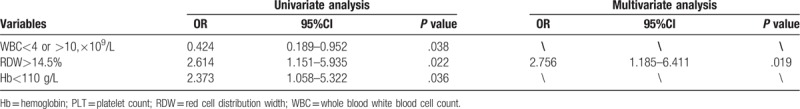
Univariate analysis and multivariate analysis GBC lymph node metastasis.

## 4 Discussions

Studies have confirmed that there is a close relationship between cancer and chronic inflammation. Chronic inflammation can damage cellular DNA, leading to repeated proliferation and repair of tissues. This process involves the release of cytokines, which makes cells susceptible to malignant transformation and ultimately to cancer.^[15,16]^ Tumor cytokine-mediated inflammation can promote tumor growth, invasion and metastasis.^[17]^ As one of the inflammation markers, RDW can reflect the progress of chronic diseases and is related to the results of chronic diseases.^[18]^ In addition, RDW is positively correlated with the markers of inflammation, such as erythrocyte sedimentation rate (ESR) and hypersensitive c-reactive protein (CRP), confirming the association between elevated RDW and inflammation, and is important for tumor progression and prognosis.^[19]^ Studies have shown that elevated RDW is associated with many poor prognosis of chronic diseases including inflammation, kidney disease, cardiovascular disease, malignant tumors and autoimmune diseases.^[20–22]^

The relationship between chronic inflammation and GBC has aroused people's attention. Repeated trauma and tissue repair caused by gallstones can cause chronic inflammation of the gallbladder, which is one of the important links in the development of gallbladder cancer.^[23]^ Therefore, we have reason to suspect that RDW elevation is related to GBC. Our results indicate that RDW levels in GBC patients are significantly higher than in healthy controls, moreover, the higher the TNM level, the more severe the metastasis, and the higher the RDW level. Correlation analysis showed that RDW was positively correlated with GBC TNM staging. ROC curve analysis showed that the optimal cut-off value of RDW in GBC patients was 14.5%, TNM stage and lymph node metastasis was statistically significant between RDW. Multivariate logistic regression analysis showed that RDW was an independent risk factor for advancements of GBC. Yang et al^[24]^ proved that RDW was associated with cancer stage in colorectal cancer patients. Similarly, we believe that elevated RDW can assess the extent of GBC progression.

The mechanism of elevated RDW in GBC patients remains unclear, but possible mechanisms include the following: Literature studies have shown ultrastructure of red blood cells changes during the inflammatory response and then participates in the inflammatory process in a synergistic manner.^[25]^ Inflammatory responses and oxidative stress can affect erythropoiesis, altering red blood cell deformation and half-life, leading to elevated levels of RDW.^[26]^ Chronic inflammation leads to increased secretion of inflammatory cytokines, such as tumor necrosis factor alpha (TNF-α, interleukin 1 and IL-6, which inhibit the production of erythropoietin (EPO) and cause an increase in RDW. IL-6 induces the expression of ferritin and further limits the iron supply of red blood cells by the action of macrophages. Besides, IL-6 can also stimulate the expression of hepatic hepcidin and inhibit the absorption of iron into the duodenum.^[20,27–29]^ Thus, an increase in RDW may indicate the presence of pro-inflammatory cytokines and tumor chemokines. Studies have shown that elevated RDW is related with the severity of chronic liver disease.^[30]^ Chronic liver disease is often associated with malnutrition, such as iron deficiency, vitamin B12 or folic acid, which affects red blood cell production and leads to an increase in RDW, suggesting that RDW is one of the body's nutritional indicators.^[17,31]^ For these reasons, we can reasonably conclude that the elevated RDW in cancer patients are associated with inflammatory factors and malnutrition. That is, as cancer progresses, the tumor-associated inflammatory response becomes more pronounced, and as the patient's condition deteriorates and malnutrition, the body further stimulates an increase in RDW, thereby explaining the relationship between elevated RDW and GBC progression.

There are some limitations in this study. First, as a retrospective study of patients with GBC, lack of follow-up and small sample size make it difficult to clarify more details between RDW and GBC. Second, although this study adjusted for many risk factors and diseases that may affect RDW levels, there may be other confounding factors. Thus, a large number of prospective studies are still needed to confirm the role of RDW in GBC.

In conclusion, our study revealed a correlation between RDW and gallbladder carcinoma, suggesting that RDW may be a potential marker for disease progression in GBC patients. Since RDW values are available through routine blood tests, this inexpensive and readily available parameter may serve as a new and convenient marker for disease progression in patients with malignant tumors.

## Author contributions

**Data curation:** Youjun Xie, Lingling Zhang.

**Formal analysis:** Youjun Xie.

**Funding acquisition:** Ling-ling Zhan.

**Methodology:** Youjun Xie.

**Writing – original draft:** Youjun Xie.

**Writing – review & editing:** Ling-ling Zhan.

Ling-ling Zhan orcid: 0000-0002-5328-1622.
